# ‘A Constant Battle for Services’: Parents' Experiences on Healthcare Services for Children With 22q11 Deletion Syndrome

**DOI:** 10.1111/hex.70911

**Published:** 2026-10-09

**Authors:** Sophie Ayoub, Sandra Meier, Cheryl Cytrynbaum, Ann Swillen, Patricia Lingley‐Pottie, Andrea Shugar, Nicolas Graf, Holly Carbyn, Abiaz Hossain, Nathan Rowarth, Bernice S. Elger, Eva De Clercq

**Affiliations:** ^1^ Institute for Biomedical Ethics University of Basel Basel Switzerland; ^2^ Department of Psychiatry Dalhousie University Halifax Canada; ^3^ IWK Health Centre Halifax Canada; ^4^ Division of Clinical & Metabolic Genetics and the Department of Genetic Counselling the Hospital for Sick Children Toronto Canada; ^5^ Department of Molecular Genetics University of Toronto Toronto Canada; ^6^ Center for Human Genetics University hospital UZ Leuven Leuven Belgium; ^7^ Department of Human Genetics KU Leuven Leuven Belgium; ^8^ Faculty of Medicine University of Basel Basel Switzerland; ^9^ Center for Legal Medicine, Faculty of Medicine University of Geneva Geneva Switzerland

**Keywords:** 22q11 deletion syndrome, access to care, equity, healthcare services, lived experience, qualitative research, rare disease

## Abstract

**Introduction:**

22q11 deletion syndrome (22q11DS) is a rare disease with a neurodevelopmental component, and the most common microdeletion in humans, that affects many systems. The diagnosis is most often made in childhood. Parents may experience a high burden of care due to the multiple medical consultations and services needed.

**Aims:**

This study aimed to examine the parental perceptions of healthcare services for families of children with 22q11DS.

**Methods:**

This interview study was a component of a broader mixed‐methods research project. The qualitative segment focused on insights from purposively selected families caring for children with 22q11DS, using semi‐structured interviews. In this paper, we report our results, following the availability, accessibility, acceptability and quality (AAAQ) framework for humanitarian settings adapted to the 22q11DS context.

**Results:**

Interviews were conducted with 40 parents of children of various ages, from Europe, Canada and the United States of America. Regarding the availability theme, parents expressed concerns about the lack of multidisciplinary clinics and specialists. Accessibility to services depended on the location of the clinics, healthcare coverage, administrative burden and access to information. Acceptability was influenced by harmful language in healthcare around 22q11DS and the lack of services. The quality of available services depended on the support of healthcare providers.

**Discussion:**

This study examined healthcare service provision for children with 22q11DS using the AAAQ framework. It identified key challenges, including limited access to specialised, empathetic providers, a lack of caregiver support, and systemic issues such as unequal resource distribution and bureaucratic barriers. The framing of (ab)normality and inadequate language further hinders equitable care. Improving service quality requires continuous support, better access to information, and education.

**Lived Experience or Public Contribution:**

Caregivers with lived experience participated in this study and shared perspectives that informed the interpretation of the findings. Representatives of patient organisations also provided valuable support and input.

**Abbreviations**
22q11DS: 22q11 deletion syndrome
AAAQ: frameworkavailability, accessibility, acceptability and quality framework
GDPR: General Data Protection Regulation
HCP: healthcare professional

## 1 Introduction

Being one of the most common microdeletion disorders, occurring in approximately 1 in 2148 live births [1] and 1 in 992 pregnancies [2], 22q11 deletion syndrome (22q11DS) occurs as a result of a microdeletion on the long arm of chromosome 22 [3]. Symptoms vary significantly between individuals and can include multiple systems. Common manifestations include cardiovascular anomalies such as congenital heart defects, endocrine dysfunctions (e.g. Parathyroid and thyroid dysfunction), immune system abnormalities (T‐cell dysfunction), neurodevelopmental, and psychiatric symptoms [3, 4, 5]. Diagnosis is often made in childhood and parents experience a high burden of care due to the multiple medical consultations and services needed [5, 6].

The literature on 22q11DS is often heavily focused on medical and clinical aspects, primarily authored by healthcare professionals (HCPs) with an emphasis on diagnosis, treatment, and physiological mechanisms, while overlooking the lived experiences of patients and their families [7, 8]. As a result, there is a growing call for research that examines the perspectives of caregivers to gain a deeper understanding of the needs and challenges faced by individuals with 22q11DS and their families.

Parents of children with 22q11DS provide a unique firsthand perspective that medical staff may not fully grasp [9]. Parents live with the daily realities of managing complex care routines, navigating multiple appointments, and dealing with emotional and familial burden [5]. Their insights reveal gaps in the healthcare system, such as delays in diagnosis due to a lack of awareness among healthcare providers [10].

Listening to parents ultimately helps healthcare professionals design more holistic and patient (and family)‐centred care plans that address both medical, psychological and practical needs. Including their voices ensures that care strategies are realistic and feasible in the context of family life. Moreover, parents' opinions can guide policy changes and resource allocation to better support families affected by 22q11DS [10].

Although some research projects have focused on the perspectives of parents of children with 22q11DS, these have either focused on one specific dimension of caregiving (e.g., research to evaluate caregiver support [11]), or been limited in the number of participants or the geographical location of the participating parents, who all lived in the same country and had access to the same healthcare system [10, 12, 13].

This international qualitative study, embedded within a broader mixed‐method research initiative on 22q11DS, aims to explore the experiences of 40 parents (from 39 families), each caring for a child aged 3 to 15 with 22q11DS, regarding the support services available to them across the US, Canada and Europe. By adapting the AAAQ (availability, accessibility, acceptability and quality) Framework, ‘a tool to identify potential barriers to accessing services in humanitarian settings’, originally developed by the World Health Organization and the UNICEF [14], to the 22q11DS context, we seek to understand better how families of children with 22q11DS perceive the healthcare services in terms of availability, accessibility, acceptability and quality.

## 2 Methods

This interview study was conducted as part of a multi‐centre, broader mixed‐methods research project (with 3 studies: a survey, an interview‐study and a coaching intervention trial study). Participants included parents of children with 22q11DS aged 3–15 years, with the primary goal of understanding in‐depth their caregiving experiences.

### 2.1 Sample

The interview study included 40 parents (from 39 families, one parent joined the other during the interview) from Europe, Canada and the US, who were caring for a child (aged 3‐15) with 22q11DS for at least 6 months. Other inclusion criteria included 1. the ability to provide written informed consent and 2. access to an electronic device connected to the internet as the interviews were conducted digitally. Language was not an exclusion criterion if it was spoken by a member of the research team (see Data collection). Initially, participants were recruited through patient networks and organisations, and social media platforms to increase the visibility of the study. 156 participants were invited to complete the survey undertaken by the research team in Canada, with the initial goal of depicting the psychosocial challenges of families of children with 22q11DS and enhancing their wellbeing (the details and results of the survey will be published separately). 92 completed the survey at the end of which, parents were asked if they were interested in taking part in the interview study and to provide their contact details. 39 parents signed up to participate in the interviews. The contact data of consenting parents was securely transferred to the European research team for follow‐up and storage on a secure university server in accordance with the General Data Protection Regulation (GDPR), via a password‐protected Excel file with the password sent separately via email.

### 2.2 Ethics Approval

The study was approved by the Ethics Committee of the University of Basel for research with human subjects. The study was conducted in compliance with the protocol, the current version of the Declaration of Helsinki, the ICH‐GCP, and ISO EN 14155 (where applicable) as well as all relevant national legal and regulatory requirements.

### 2.3 Data Collection

39 interviews (in one interview, 1 mother and father were interviewed together) were conducted between September 2023 and June 2025. The anticipated sample size of approximately 40 participants was determined a priori based on the principal investigator's previous experience conducting qualitative research in families affected by rare genetic conditions. Throughout data collection, the research team continuously monitored the exploration of new themes. While we observed that thematic saturation had largely been achieved after approximately 36 interviews, three additional families had already expressed interest in participating and were subsequently interviewed, enriching the existing findings. Once the first author, SA, received the contact details of parents who agreed to participate in the interview study, she reached out via email to schedule interviews. No prior relationship existed between the two parties before that. The participants were given an information sheet and a written informed consent to sign before the interview. Those with clarification questions were invited to ask them via email or over Zoom. The interviews were all conducted via the digital platform Zoom by SA (*N* = 37, 4 in French and 33 in English) and EDC (*N* = 3, 1 in Italian, 1 in Dutch and 1 in English). SA is a female medical doctor working on her PhD project and EDC is a senior researcher in bioethics. Both are trained to conduct qualitative research. The interviews lasted between 40 and 102 min. First, the purpose of the study and the minimal risks were explained. Respect for privacy and confidentiality was explained prior to starting the semi‐structured interview. The interview guide was based on existing literature and shaped through discussions within the research team (see Supporting Material). It consisted of questions and prompts focusing on parents' caregiving experiences, the challenging and rewarding aspects of caregiving, parental needs, availability and accessibility of support services, and the perceived diagnosis experience and their perceptions of the future.

### 2.4 Data Analysis

After approval from the participants, all interviews were recorded via Zoom on the interviewer's local computer at the University of Basel. They were then transcribed and coded using the qualitative analysis software MAXQDA 24 to support structured data analysis. Following verbatim transcription, potentially identifying information about the participant was removed from text to maintain respect for privacy, confidentiality and anonymity. The interviews were transcribed by the main researcher, SA, and an assistant researcher, NG, either manually using MAXQDA or using ADOBE Pro. One interview was coded by EDC and SA, with most codes identified, two interviews with NG and the remaining 36 were coded by SA. For the analysis, the authors followed a deductive coding approach guided by the AAAQ framework [14]. This framework is a conceptual model frequently used to assess rights‐based service accessibility. It provides a structured approach to interpret barriers that impede access to healthcare by analysing four key aspects: availability of healthcare facilities in sufficient quantity, accessibility, acceptability of healthcare services, and quality of said services. The framework was adapted to the context of services and caregiving experiences for children with 22q11DS. Although the coding was primarily deductive, space was left for emergent subcategories within each domain, allowing for participants' perspectives to inform more nuanced interpretations. Coding consistency was ensured through audit sessions (with SA, EDC and NG) and discussion of discrepancies until consensus was reached [15]. To facilitate transferability, the authors tried to give a clear description of the findings and the characteristics of the participants and illustrated them with appropriate quotes.

## 3 Results

We interviewed 40 caregivers (35 mothers, 3 fathers, and 1 mother and father interviewed together), who were taking care of children with 22q11DS aged from 3 to 15 years. The participants lived in Europe (*N* = 18), Canada (*N* = 6) or the United States of America (*N* = 16).

The majority of children were diagnosed after birth (11 in the first year) and 4 were diagnosed prenatally. Six of the children were only children, 25 had older siblings, and 12 had younger siblings. Twenty of the interviewed parents had a full‐time job, 7 worked part‐time and 12 were unemployed. None of the interviewed parents was diagnosed with 22q11DS (see Table 1).

**Table 1:** Characteristics of the main caregiver and the child with 22q11DS.

FAM	Parent	Child	Age	Age of diagnosis	Siblings	Civil status	Professional status
FAM1	Mother	Male	15to16y	7 y	2	Married	Unemployed
FAM2	Mother	Male	8 y	4 y	3	Married	Unemployed
FAM3	Mother	Female	4 y	2to3y	2	Married	Unemployed
FAM4	Mother	Female	14 y	6 y	None	Married	Unemployed
FAM5	Mother	Male	4 y	In utero	1	Married	Full‐time
FAM6	Mother	Female	11 y	2 months	1	Married	Part‐time
FAM7	Mother	Female	10 y	In utero	None	Married	Full‐time
FAM8	Mother	Male	13 y	2 y	1	Married	Part‐time
FAM9	Mother	Male	5 y	In utero	3	Married	Unemployed
FAM10	Mother	Male	5 y	6 weeks	1	Married	Full‐time
FAM11	Mother	Female	11 y	5 y	None	Married	Full‐time
FAM12	Mother	Male	9 y	6 to 7 y	2	Married	part‐time
FAM13	Mother	Female	10 y	1 to 1.5 y	1	Married	Full‐time
FAM14	Mother	Female	15to16y	5 y	2	Married	Full‐time
FAM15	Mother	Male	9 y	7 y	3	Married	Unemployed
FAM16	Mother	Female	8 y	5–6 weeks	1	Married	Part‐time
FAM17	Mother	Female	7 y	3 y	1	Separated	Full‐time
FAM18	Mother	Female	15 y	8 y	1	Married	Full‐time
FAM19	Father	Male	9 y	7 months	1	Married	Full‐time
FAM20	Mother	Male	15 y	3 y	2	Married	Full‐time
FAM21	Mother	Female	4.5 y	2 y	None	Married	Full‐time
FAM22	Mother	Female	12 y	5 y	1	Married	Unemployed
FAM23	Mother	Male	15 y	4 y	2	Divorced	Almost Full‐time
FAM24	Mother	Male	5 y	Afew weeks	2	Married	Part‐time
FAM25	Mother	Female	7 y	4 y	3	Married	Full‐time
FAM26	Mother	Female	8 y	4 weeks	1	Married	Unemployed
FAM27	Mother	Female	11 y	In utero	1	Married	Unemployed
FAM28	Father	Male	10 y	4 y	2	Remarried	Full‐time
FAM29	Couple	Female	13 y	1 month	1	Married	Unemployed
FAM30	Mother	Male	8 y	7 y	1	Married	Unemployed
FAM31	Mother	Male	15 y	7 y	1	Married	Part‐time
FAM32	Mother	Female	15 y	10 days	Many	Married	Full‐time
FAM33	Mother	Female	7 y	7 y	1	Married	Part‐time
FAM34	Mother	Female	11 y	5 y	None	Single	Full‐time
FAM35	Mother	Female	5 y	Afew days	3	Married	Unemployed
FAM36	Mother	Male	15 y	4 month	1	Married	Full‐time
FAM37	Mother	Male	11 y	9 y	2	Married	Full‐time
FAM38	Father	Female	2 y	1 y	1	Married	Full‐time
FAM 39	Mother	Male	10 y	3 months	None	Married	Full‐time

Abbreviations: FAM, family; y, years.

We followed the AAAQ framework to report our results after adapting it to the settings of healthcare in the context of 22q11DS with four themes (availability, accessibility, acceptability and quality) and many sub‐themes (see Figure 1). Results on each section were reported: 1. Availability of medical services, 2. Accessibility in terms of physical (location), financial (costs and resources), administrative (administrative procedures), social (social barriers) and informational, 3. Acceptability focusing on the respect of the person regardless of age and special needs and 4. Quality of available medical services, specifically those provided by HCPs. Illustrative quotes were included in tables, each table for a sub‐theme.

**Figure 1:**
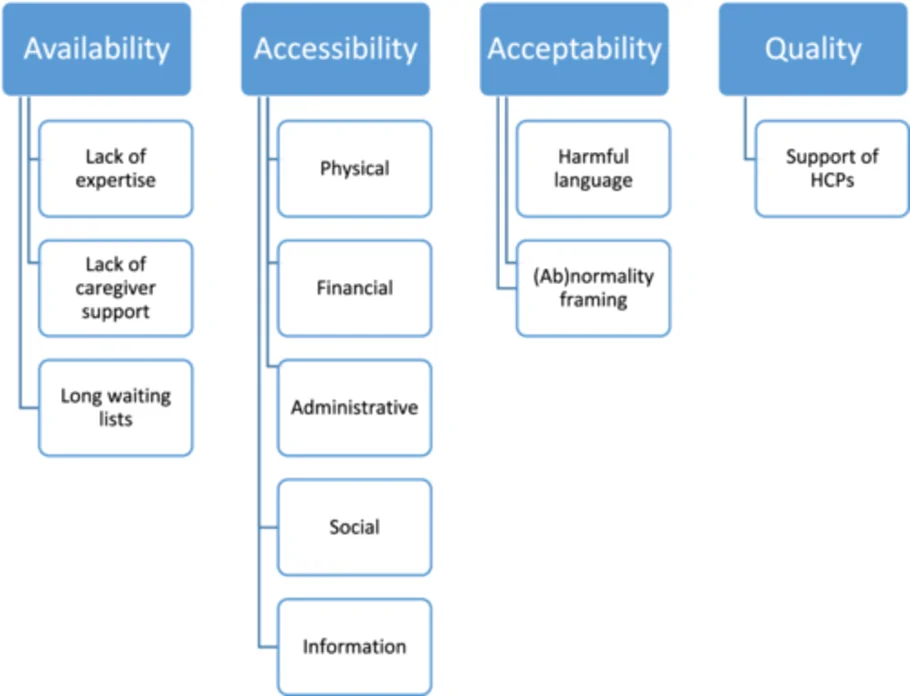
Themes and sub‐themes on healthcare services for families of children with 22q11DS.

### 3.1 Availability

Based on the parental accounts, there was a great variation of parental experiences of available healthcare services even within one country.

#### 3.1.1 Lack of Services and Expertise

Most interviewees considered experienced healthcare providers to be an essential element in the caregiving process. The HCP's awareness of 22q11DS significantly influenced the input they brought to the care process. This manifested in the way professionals handled the medical challenges and whether they treated the child similarly to other patients without acknowledging the impact of 22q11DS.

As individuals grow, their developmental needs change, but this progression is often not adequately addressed. As a result, parents are left navigating a complex system, caught between paediatric and adult services that may not align with the evolving needs of their child. The lack of services was, for example, described by one participant as ‘a matter of lottery’. According to a few participants, paediatric psychological care was particularly unavailable due to the high demand of psychological services. In one example, accessing psychological support, even virtually, was not possible if the expert did not live in the same area (see Table 2).

**Table 2:** Availability (Lack of services and expertise).

Parent 1 (Mother, Europe)*: ‘A paediatrician is so specialized, especially the one I see, because she knows so much about 22q and special needs and my GP (general practitioner), they're great when you have an infection or so but they have no clue about 22q, at all!’*
Parent 34 (Mother, US): *‘There was no 22q clinic at the time. I think a clinic finally opened up like years later. I think, probably like 2 or 3 years after she got diagnosed, because they finally saw that there were enough kids around here that had it. So yeah, it (having a clinic) would have made a difference as to how I would approach her from the beginning.’*
Parent 14 (Mother, Europe): *‘The psychological side that's difficult because like I said, it's difficult to know what to say to her, it's difficult to know how to handle it and trying to get psychological help here in (location) since the COVID epidemic has become incredibly difficult because there are so many young people who need help.’*
Parent 32 (Mother, US): *‘And they have it (psychological support) in other states. But you can't cross state lines to do virtual appointments, you can do it if it is only within your state. So I could take her out of state, but I would have to physically take her there, I would have to drive her there*.
Parent 28 (Father, Europe): *‘And then it's a lottery system. So every year, there are X amount of free spots and then they organise a lottery, they pick children from the list. It (getting support) is a matter of being lucky.’*
Parent 4 (Mother, Europe): *‘I think up till now her sort of medical challenges have been dealt with quite well because she's been on the paediatric team. But when she had this medical issue (the heavy menstruation) they don't deal with necessarily as a paediatric issue. So I found myself in between paediatric and adult care and it's been an absolute nightmare because if you're trained as an adult doctor you can't deal with children. So even just to have blood taken, they were like: ‘Oh no we can't take blood because we're not trained to take blood from children.’ So it's been quite stressful because there is no joined up, integrated approach and I'm struggling to find an appropriate approach.’*

#### 3.1.2 Lack of Caregiver Support

When asked ‘what's missing’ in the available support services, some of the parents emphasised the importance of comprehensive caregiver support, specifically psychological support. Many caregivers felt that the latter mainly targeted the child and not the family, particularly the parents, who also live with the daily challenges of 22q11DS. The few interviewees who had the opportunity to speak with a psychologist, to guide them through parenting a child with 22q11DS, emphasised the positive impact of these encounters on their mental health and well‐being. One parent, for example, expressed a desire for their psychologist to have a better understanding of their caregiving challenges. Consequently, they would not waste their time explaining 22q11DS, but discuss the impact it has on them and their personal struggle (see Table 3).

**Table 3:** Availability (Lack of caregiver support).

Parent 12 (Mother, Europe): *‘But as I said, we've had no kind of psychological support for the whole family. Like we've never had a family counselling kind of thing.’*
Parent 16 (Mother, Europe): *‘And the only thing that we actually miss is a psychologist that has a knowledge of 22q11. There can be a lot of pressure and strain on us and you don't want to talk to someone (who does not understand) and have to explain what you're experiencing with your child. It would be super that we could find, in (country), a psychologist that has knowledge about (the daily challenges of) 22q11 so that you just can go to them and really talk about yourself instead of about your child.’*
Parent 17 (Mother, Europe): *‘At the Genetics Institute (name of clinic), there is also a psychological unit. So they can help children, people with the disease, or parents. So I went to see them. I consulted this psychologist several times for myself, to talk about it and get it off my chest.’*
Parent 26 (Mother, Europe): *‘What I found with the child psychologist last year was that she was the link that was missing, between me parenting a child to 22q and navigating the whole thing, the challenges, the worry. Am I doing the right thing? What should you know? Just the tiny bit of parental guidance that's needed in parenting any child with additional needs (…) Even though it's called child Psychology Services, I felt, I mean, you know, it's for me, it was all about helping me to know what the right and wrong thing to do.’*

#### 3.1.3 Long Waiting Lists

Most of the participants acknowledged that the waiting lists for needed services were too long (particularly for psychological care), reaching years of waiting in some cases, even when related to urgent matters such as psychological distress that could endanger the life of their child. The waiting lists were lengthy in almost all geographical locations. Some interviewed parents, however, seemed to appreciate the availability and quality of these services regardless of the waiting time. For some medical specialties, access to urgent care was faster (i.e. endocrinology) (see Table 4).

**Table 4:** Availability (Long waiting lists).

Parent 1 (Mother, Europe): *‘So CAMHS (Child and Adolescent Mental Health Services) that we have in the (location), they're very hard to get. The waiting list is a 2‐year waiting list to get on but when you're there, they're absolutely amazing, absolutely brilliant.’*
Parent 14 (Mother, Europe): *‘The psychological side has been the side that's really difficult to access. I know all the others I could ring their secretaries and she could be seen the day after tomorrow if I was concerned about something. Whereas we've asked for (psychological) help many times and I find this quite incredible, I mean she’ s on the list to be helped but that's a year long wait. The only way that she would be moved up the list and this just makes me so sad if she actually tries to take her own life!’*
Parent 28 (Father, Europe): *‘For example, a personal budget, that you can request but it's a process of first, you to know that you are, entitled for the personal budget and that's a budget I would receive, like, a monthly amount that as a parent can then spend for activities and support. But the first step is requesting it and that takes like 2 or 3 years, and then you're on a waiting list to actually get the budget. And it's like again, 4 or 5 years. So it's a very, very long process (…)*
Parent 31 (Mother, US): *‘The other piece and I would say that in terms of services, it's just waiting lists. Everything is a waiting list.’*
Parent 36 (Mother, US): *‘Especially the worst one is neuropsychology. He needs an updated exam for his ADHD. And we've been on the waiting list for 2 years, to try to get it done. Therapists are the hardest to get by far. Endocrinology is pretty good. He actually fits us in pretty quickly because the thyroid needs immediate attention.’*

### 3.2 Accessibility

Accessibility was defined by many following aspects: physical, financial, administrative, social and by access to information.

#### 3.2.1 Physical Accessibility

Physical accessibility refers to the location of services (within a reasonable distance). Our results showed that accessing services in this context was complicated and one of the solutions that helped some parents was accessing them in school or through the local service network (as opposed to the big expert centres).

The majority of caregivers found physical accessibility to services difficult. Travelling long distances to access specialised services and spending hours on the road was time‐consuming and a huge burden on parents especially when combined with the multiple tasks they needed to fulfil. Those who did not have a specialised clinic in 22q11DS healthcare services nearby experienced a greater burden.

Additionally, depending on the health and education systems in each region, certain medical services may be offered during school hours. The child with 22q11DS could then have speech‐language therapy or occupational therapy as part of their school schedule. This eased the burden on parents, particularly in terms of time management (see Table 5).

**Table 5:** Physical accessibility.

Parent 20 (Mother, Europe): *‘But the (clinic) helped us then (…) They wrote to the local CAMHS (Child and Adolescent Mental Health Services), and got them to take him on. And then discharged us from (location). Like, it was two‐and‐a‐half‐hour journey. It's a whole day. And it's a long day with him to get there. So now we are seen locally for that.’*
Parent 26 (Mother, Europe): *‘But it meant taking her out of school, driving her to this place every Thursday at a time. And, you know, if you're working full time, that's complicated. Her needs are not complex physically, but they are complex in every other way.’*
Parent 31(Mother, US): *‘Unfortunately, we don't live near a 22q clinic. We'd have to travel pretty far for that. So we see them virtually. There is a 22q clinic in (location) at the (clinic) medical centre, but they don't take our insurance (…) So I have to sort of be the gatekeeper and then try and set up appointments and life is busy, and it's hard to travel 5 h to go see them all. And their schedule is very busy. So it's tough to manage appointments.’*
Parent 35 (Mother, US): *‘So we don't have a 22q clinic locally. I know that there's one in (location), which is about 2 h away. We have not been there, we just have always been able to get, all the specialists covered in our local children's hospital. It's pretty good. So we haven't been to the 22q clinic. We've thought about it, but it's just far away.’*
Parent 16 (Mother, Europe): *‘So at her school, there's this kind of multifunctional thing where she gets physiotherapy and where she gets speech therapy. And we did that for six years with private caregivers. But now it's at school. So that's a relief for us, because it's during the day and we don't have to hurry to get to the one of our other appointments.’*

#### 3.2.2 Financial Accessibility

Financial accessibility refers to the funding of the services, their costs and whether the latter are manageable or reasonable given the economic situation of the family. Healthcare services were either publicly or privately funded depending on the location as healthcare coverage in the US is primarily based on private insurance and sometimes on public programmes, whereas most European countries and in Canada provide universal coverage through publicly funded systems.

##### 3.2.2.1 Public Services

Almost all participants voiced concerns about the lack of an appropriate financial support system. The financial need increased depending on the medical and daily needs of the child with 22q11DS. The public health system was either directly financed by the government or through an insurance system (i.e. Medicaid in the US). The eligibility criteria varied among countries and states. Some eligibility systems were based on the parents' salaries or their employment status, others on how much they spend financially for the 22q11DS child's needs and others were based on prerequisites such as an official medical diagnosis of the condition or an HCP's prescription. A few parents were offered a carer allowance if they were unemployed, but the amount was considered insufficient to even ‘survive’. Additionally, one parent explained that in their area, you get public funds depending on how much you invested in your child's care, so this family, for example, got less funding when they stopped visiting private services.

##### 3.2.2.2 Private Services

In some cases, families had to pay privately for services. This happened when they were deemed ‘too rich’ to receive governmental help, or when the medical support offered by public services (i.e. speech‐language therapy sessions) was insufficient for their child to improve. This was also the case for the caregiver support services (i.e. psychological support) that were funded privately by a few parents (see Table 6).

**Table 6:** Financial accessibility.

Parent 4 (Mother, Europe): *‘So have you heard of DWP? Which is the ‘Department of Works and Pensions’. So they deal with the cares allowance. You have to put all the paperwork in and because I don't work I obviously then get the carer allowance. I mean it's nothing it's like 52£ a week. I mean how can you survive with 52£ a week, I have no idea.’*
Parent 7 (Mother, US): *‘Whether it's health related, mental health, educational, they just need more support. And maybe even more support through like (clinic) … I can't even get (name) on Medicaid. There's certain programs out there that she would probably benefit from. Like counselling for sure.’*
Parent 16 (Mother, Europe): *‘So it works with points and the more points you get; the more financial resources you get. There are three pillars. And the third one is based on how much you as a parent put into it. And these points are tripled. And due to the fact that we went to private caregivers, the points in that pillar were very high. And therefore we got more resources. Now that she's going to the school and to the special centre, well, that pillar doesn't count as much anymore. So the resources are lowered this year.’*
Parent 18 (Mother, Europe): *‘Because she had the surgery on the palate, the responsible surgeon wrote a prescription, and with that prescription we pay less. So that's covered by the government, indeed, by the social security. And the same for the physiotherapy that she followed. As long as you have a prescription from a doctor or a physician, then you get some money from the government.’*
Parent 38 (Father, Canada): *‘There is some funding for kids with severe disabilities in (location) but it's income based. And I'm above that income threshold. And so, I'm penalized.’*
Parent 13 (Mother, Canada): *‘There is support out there. Like there's like that resource group for us here in (city), that resource for such exceptional children. There's a special service at home and other support services but I don't know them all because some of them are based on your salary that we don't qualify for (…) Like we paid for private therapy three days a week (…) I mean we have good jobs but still was a lot of money. But then I was thinking about parents that really maybe don't have any insurance or can afford private like how fair is that!’*
Parent 27 (Mother, US): *‘Price obviously being a big factor for us. We do get some financial assistance through the hospital system. But we don't qualify for governmental programs because my husband makes too much money. But I had to quit my job, to take care of her. And that has been a, a huge financial burden on the family. Because, you know, the reality is we kind of live in a two income world so of course, it's hard, we're in debt. And, I've already had to cash in ten years of savings to help us get by, an, we're about to have to cash in the rest of my life savings to pay this off (.) One of the things they count is how much you're taking out of your savings to pay your bills. So I think that actually is going to help us. And I think we'll qualify for that then.’*
Parent 28 (Father, Europe): *‘To get the diagnosis, it's mainly a cost thing. Like you need to pay the money and then you normally get the diagnosis which is actually not (medically) necessary. But with these papers from the doctors and with the diagnosis, you can put yourself as a candidate on the list for a special school (…) A quite expensive diagnosis, but it's like your ticket in which you cannot go to that system.’*
Parent 31 (Mother, US): *‘I know there are programs you can apply for that are state or government funded. I don't think we would qualify. From what I've looked into, your income has to be really low, like poverty level. And so from what I've looked at, we're not eligible for it.’*
Parent 32 (Mother, US): *‘There is no publicly funded anything here. So it's all private. And we have fortunately, we have a very good insurance. It's just the, the waiting lists are really long and then I could pay for it out of pocket. But, you know, it's $300 an hour (…) that's a lot of money.’*
Parent 31 (Mother, US): *‘So (child's name)‘s just is on this specific, state funded insurance, the rest of the family is not. And our insurance is terrible and super expensive. So I don't get a lot of health benefits, like, as far as mental health benefits, unless I want to pay an insane amount of money.’*

#### 3.2.3 Administrative Accessibility

Administrative accessibility refers to the procedural steps that must be completed to access healthcare services. The procedural steps to access care services for children with 22q11DS and their families were complicated for the majority of parents. There were too many documents to provide, too many battles to ‘fight’, and a lot of getting ‘lucky’ to enter the system that provides adequate services. Moreover, one parent questioned why these forms should be filled by the direct caregiver. She felt vulnerable and she found it difficult to fill out the documents because the questions highlighted the disabilities and abnormalities of her child, whereas she, as a mother, sees her child as perfect.

Furthermore, having an official diagnosis, for 22q11DS or for a specific related disorder (i.e. for intellectual disability), made services more accessible: some interviewees were aware of the need to pursue additional medical tests that might not be needed for therapeutic endeavours, but only for administrative purposes. These were necessary to document officially the need for certain services, for example, speech‐language therapy (See Table 7).

**Table 7:** Administrative accessibility.

Parent 4 (Mother, Europe): *‘I think the problem with support over here: it's a fight to get it. Everything's a fight. Everything is paperwork. You've always got to gather your information. You've always keep all the files. You've always got all the medical notes.’*
Parent 6 (Mother, Canada): *‘So I called the hospital and the woman said: ‘Well she (your daughter) is not on my case load. So I can't help you.’ And I was like: ‘Well we live here now and she has a G‐tube and someone's got to help us.’ And so it was really hard to access care. And that's when I reached out to like the hospital administration and I was like: ‘This just happened (…) this woman was just some nice lady doing her job and my daughter wasn't on her case load but the fact is I couldn't get help, we couldn't access care, that's the problem.’ So anyway they really need a 22q clinic with coordinated care. Because all the many pieces that (name) needs, that's like me asking for the referrals and then following up and calling the clinics.’*
Parent 11 (Mother, US): *‘They (the HCPs) kept saying she's not growing but nobody could give me an answer why (…) And I think definitely if we had that test before (…) then soon as she was born, we could have had specialized bottles (for feeding), we could have had just so much more resources, I believe. And even when she wasn't talking, we have like resources for children and we kept getting denied. They kept saying she's fine. And even after she got this mucosal palate issue, it was a fight with them. I was citing law and everything with them, because they were saying, she doesn't need services. And I'm like she doesn't talk, she needs services (…) So it's been a lot of fighting.’*
Parent 26 (Mother, Europe): *‘I got great advice from the early intervention nurse coordinator who said, look, these forms are horrible, they're awful, they're not nice to have to fill out anyway, but you're going to have to make it a paper exercise only, compartmentalise it. And for that hour that you're doing the paperwork, you have to detach yourself from the beautiful child that you have and think about all of the difficulties that that child has, all of the limitations and all of the challenges. And it's not about exaggerating, and it's not about lying on a form, because that's illegal, but it is about being realistic and not looking at it through rose tinted glasses. And that was incredibly difficult (…) They (the forms) have to be filled out by the guardian or the parent which is unfair (…) You know as a new mum (to fill these papers), the vulnerability is huge.’*
Parent 28 (Father, Europe): *‘The places are very limited. So it's, on one hand, you need to have the correct papers, so you need all the reports from the doctors. For him, it was still quite defendable because he has this genetic disorder. You could really pinpoint like, he has this. So therefore we know that these will be issues (…) But you also need to put effort and work to make sure that you have the right papers and you have the rights into specific systems because everything takes very long.’*
Parent 23 (Mother, Europe): *‘For instance, some of the 22q parents, they tell me they took the test and: ‘Oh, my daughter is 12 but in behaviour she's like four’. I never took those tests (…) what does it help me if I know that he's not really 15, but he's like 8? ‘but maybe in the end, I will have to get those arrangements done because otherwise they won't give me any help.’*
Parent 29 (Mother, Europe): *‘(…) she's given these extra diagnoses of intellectual disability and autism. Didn't matter about 22q. That got us nowhere. But it took these subsequent diagnoses to get a child plan at school, to get a multidisciplinary and educational psychologist involved. And they never referred (name) to educational psychologist up until now. So that side of things has been hard and we've put in so much. Well we've tried to do everything we can (privately) to fill in the gaps.’*
Parent 30 (Mother, Europe): *‘When he started his first year in school, he had his speech language report. So he was getting support from speech and language after that assessment. Then when he got his dyspraxia reports, he was getting help on motor skills for that year. And then the following year, he got the 22q diagnosis. So that's when they put more support in there.’*
Parent 34 (Mother, US): ‘*Then finally when she was diagnosed with 22q11DS, they have what it's called for special education children, they developed a program for her to see what she needed. They got the psychologist involved and whoever else involved to see what she needs, so they put her in speech therapy classes and somewhere along the line she got diagnosed with intellectual disability so they put her into another program.’*
Parent 37 (Mother, Canada): *‘(…) I'm lucky in a way, that's what I get for these services, because I've already entered the system for something else (another congenital condition of her child). And if I didn't have something else, I don't know if I'd really have so many services.’*

#### 3.2.4 Social Accessibility

Social accessibility refers to respect and non‐discrimination in the provision of health services. We were able to identify one particular area where a breach of respect occurred: the oversight special needs. A few interviewees observed that children who functioned at a younger developmental level compared to their age were expected to meet standards they could not realistically achieve. In some cases, parents reported that support was only provided when a child's needs were very severe, leaving those with moderate needs overlooked. This was mentioned in regard to respite care that came at a price and was only possible to obtain through a charity organisation. Several parents also questioned whether those who did not speak the main language used for service delivery might be excluded, as the language level used to help them was too advanced. According to a few caregivers, even when services were available, they were hard to access without expert knowledge, persistence, or social support (see Table 8).

**Table 8:** Social accessibility.

Parent 1 (Mother, Europe): *‘So this is a benefit, a personal independent payment benefit and if you have disabilities you're entitled to apply for this. We did the usual chitchat and she (a HCP) is so good with (name) (…) She could just see it from all angles (…) and actually she put several things in place for when he is moving forward into young adulthood, to the disability care program, learning disability team and social care team, and then wrote this incredible letter that meant that we got our benefit without even a question.’*
Parent 12 (Mother, Europe): *‘It would affect your mental health as a parent, having this constant battle for services that aren't common (intellectual disability) (…) unfortunately here in (country), we haven't fully implemented the UN Convention of the Human Rights, you know, in relation to health care. So, (name) would have a right to a diagnosis. He doesn't have a right to intervention and therapy.’*
Parent 20 (Mother, Europe): ‘*They're not able to coordinate their care. I struggle to coordinate his care and the level of paperwork, I struggle with doing it in my job all the time and having pretty good English. I don't understand how people cope if they don't have good literacy or good English.’*
Parent 26 (Mother, Europe): *‘No there isn't. There is no respite care for the kids with severe physical, and complex needs. Never mind someone like (name). She doesn't have those immediate physical demands. So respite would come at a cost. And if I decide to go away for a weekend, we have to hire someone and stay with the girls, which is standard babysitting (…) But, we did get referred to a charity who provide nursing care for children with medical needs. So we go 9 h a week of respite care while she had the NG tube. And that was fantastic. That kept me sane for that entire first year.’*
Parent 26 (Mother, Europe): *‘But, I think that they definitely don't get treated well, in general. And I find the more profoundly they're impacted, the more understanding people are. I think that most of our kids are in this mid‐range. And they still need support but they look like they might have a low need of support. They don't have high needs. I think the kids that have high needs of support get treated better.’*

#### 3.2.5 Information Accessibility

Information accessibility is defined by how the availability of services is communicated to the family.

The majority of participants emphasised the importance of receiving sufficient information to guide them to the necessary healthcare services throughout their life cycle. However, information was either lacking or, if available, it was often difficult to access and required specialised skills to obtain. Many found themselves having to search for this information on their own and felt that they did not get the care they deserved, but what they were ‘able to figure out’. They frequently relied on friends or healthcare providers to help them navigate the system. Despite these efforts, they did not feel supported by the healthcare system itself. This lack of institutional guidance created additional stress and made accessing essential services more challenging. One parent, for example, recommended specific guidelines for 22q11DS, for the parents, in the form of a check‐list or a tool for general needs and specific ones for special needs (see Table 9).

**Table 9:** Information accessibility.

Parent 7 (Mother, US): *‘And then making sure that that parent understands what they need to be looking for (…) So maybe there should be more like guidelines (…) like by age one if the kid is nonverbal you need to be evaluated for this and that (…) And actually they probably all just need PT, OT, speech, and audiology and cardiology. If you have 22q you need to have all these evaluations done. Pack a box off, make sure there's nothing going on. If there is, then you continue with those services as needed.’*
Parent 16 (Mother, Europe): *‘Well for us, at the moment, there are no needs I think. We already get the support of the government. In the beginning, I didn't know where to find the support. But once you know where to go, then everything is taken care of. And I'm maybe also lucky that one of my friends, she is in the health care system in (location), so she knows her way around, she knows her way around all the paperwork and the support you can get (…) And I also think that the first time you get the diagnosis, there should be some kind of leaflet, that parents receive with all the information on what support they can get.’*
Parent 28 (Father, Europe): *‘The caregiving, it's well organised in (country). So there is a lot of support. And it's also financially well organised. But the main hassle is finding your way to the right support. So there is not a handbook or a manual like who should you contact, where to look for information, where can you request for support? So that's for me very strange because you really need to browse the internet for people, figure out how to get extra support or how to make sure you get financial extra support. And the better you do the search, the more (services) you get. So that's a little bit strange about the system, it's not like you get what you deserve or what your right is. It's more like you get what you figure out.’*
Parent 32 (Mother, US): *‘I think all the information is out there. It is. But you have to, as a parent, go out and find it, I guess. And obviously I've been well educated and I made it my purpose to like get that information. But is it easily accessible? It's not. So I feel bad for other parents out there whose children have this diagnosis and they don't have the skills or the means to go out and learn things.’*
Parent 35 (Mother, US): *‘I did it all by myself. Yeah, it was a lot. That itself probably was a full time job, especially the first couple of years. But I did it all on my own. Unfortunately, the health care system is not set up really to give you much help in that regard. You have to find it on your own. So sometimes you'll look and you'll find the good things, like social worker in a hospital or something like that, and sometimes you won't (…)’*

### 3.3 Acceptability

Acceptability is defined by how the services are respectful of the individuals and families and whether they are age sensitive. In our study, we identified a harmful language problem and an (ab)normality framing.

#### 3.3.1 Harmful Language

The majority of interviewed parents emphasised that HCPs must be mindful of the language they use when communicating with them. Some of them noticed that the use of stigmatising, insensitive, or even outdated language can unintentionally cause emotional distress and undermine trust in the care relationship. In one example, the parent experienced her child being called funny‐looking and mentioned that this language was not uncommon, and that many parents see themselves in the same situation (see Table 10).

**Table 10:** Acceptability (Harmful language).

Parent 2 (Mother, US): *‘I think just having a sensitivity I would not say funny‐looking. There are some videos that I found online of similar things happening in interviews with parents. As they learn more about the syndrome and the way that facial features manifest themselves I would not use unkind nomenclature. I would never call a child funny‐looking.’*
Parent 12 (Mother, Europe): *‘And I think clinicians need to be aware of the importance of language. That's that consultant who keeps referring to it as DiGeorge. It's very irritating, because to me, the feedback I get from her continuing to call this DiGeorge is she's not up to speed on the international literature, it's called 22q (…)’*
Parent 36 (Mother, US): *‘And so I'm telling her (a HCP) all that history and she's like, yeah, that happens a lot when there's something wrong with the foetus. And I was like, wrong!! Like, that sticks in my head to this day, and I know what she meant in my head. But like, it still sticks there, like, I don't think I even paid attention to anything that she said after that.’*

#### 3.3.2 (Ab)Normality Framing

According to several parents, HCPs often emphasise abnormalities and focus on deficits when describing a child's disability, which can come across as pessimistic and disheartening. This clinical framing may overlook the emotional bond and unconditional love that parents feel for their child, regardless of diagnosis. For many families, their primary concern is not success as defined by society but the happiness of their child. When providers focus solely on limitations, it can invalidate the parents' experiences and contribute to emotional distress (see Table 11).

**Table 11:** Acceptability ((Ab)normality framing).

Parent 4 (Mother, Europe): *‘I can always remember seeing an educational psychologist (…) she was quite young and she was quite offensive in what she was saying. I can always remember her just saying, something in line of what are my hopes for (name). And really all my hope as a mom is that she has a lovely life, whatever she wants to do (…) That was my mindset but before I could even answer she just turned around and said: ‘Because she won't be normal like other children. You do realize she's going down a different path and you need to prepare yourself. She's never going to be a doctor or a dentist’ and I was like: ‘Oh my goodness, I realize that but that's not my hopes and desires for (name). My hopes and desires for (name) is just to be happy (…)’*
Parent 36 (Mother, US): *‘I remember one (HCP) in particular, he (the child) was still a baby and we brought him in because he just wasn't feeling well (…) It was our regular doctor's office, but not our regular doctor. And, so we're talking to him, we're saying, yeah he has this 22q and the doctor literally was not willing to see us. And we had sometimes doctors that don't understand his anxiety and think that he's just being dramatic. And he barely complains about anything, like his foot could be falling off and he wouldn't be complaining about it. But he actually gets anxious in a doctor's office.’*

### 3.4 Quality

The quality of services offered refers to whether they received an acceptable standard of care. It also encompasses the manner in which people are treated when accessing services. In this study, the quality was often related to the HCPs' support.

Healthcare providers play a critical role in shaping the experiences of families of children with disabilities, and their level of engagement can significantly impact the quality of care. Some parents reported instances where providers were dismissive of parental expertise in their child's condition, missed important diagnoses, or treated the child without fully considering their underlying condition, leading to a sense of being overlooked. A big group of interviewees also mentioned that they were not always listened to, and that HCPs tended to disregard their personal lived experience and their expertise of ‘their child’. In contrast, when providers were supportive, parents described feeling more confident and reassured in their caregiving roles. Positive experiences were considered a ‘big victory’ and included access to knowledgeable specialists, and timely psychological support. One parent encountered a major challenge of high turnover of specialists, which disrupted continuity of care and made it difficult to build lasting, trusting relationships with providers (see Table 12).

**Table 12:** Quality.

Parent 12 (Mother, Europe): *‘And I suppose the final thing then is obviously listen to parents. We know our children inside out. We know when there's something going on, they're not. We know this typical illness for another child is fine, for our child it's different. You know? if I hear once more he's got an atypical presentation or something, I'm going to… ‘Yeah, that's 22q.’ Nothing is typical in 22q. Even the typical presentations present atypically, you know? So… we know this as parents. We do the deep dive. We do the research. So when we say something, don't dismiss us.’*
Parent 21 (Mother, Europe): *‘I wasted time, I wasted a lot of time finding the right professionals because as parents, we often talk about this. We can tell within the first 5 min whether we're wasting our time with a professional or not, whether it's good or not, so to speak. We can tell within the first 5 min whether we've wasted our time or not and whether we'll come back or not. But for us, it's already a big victory when a healthcare professional knows about 22q11.’*
Parent 26 (Mother, Europe): *‘I remember the first summer when she was on the NG‐tube. I emailed the neonatal paediatrician, said, look, I need some help here. I can't cope with this. I need to speak to somebody for myself. So she referred me, and I got an appointment within 2 weeks with a neonatal psychotherapist. And both my husband and I attended together and separately and that was really, really good.’*
Parent 24 (Mother, Europe): *‘And then a year ago, we had our first appointment with the health service. So in order to do that, I had to take him off the disability network and put him back into the community service. And then my referral was backdated from when he was a baby, so we were seen immediately. The professionals that we've met there are absolutely lovely, but they all get promoted or transferred. So we're on our fourth one in there. They're lovely, but it's pretty disappointing that he builds up a relationship with that person, and then they leave.’*

## 4 Discussion

This study aimed to explore parental experiences on healthcare services for families of children with 22q11DS following the AAAQ framework. While originally developed for humanitarian health settings, we adapted the AAAQ framework to critically assess healthcare service provision for children with 22q11DS and their families. Both contexts (humanitarian and rare diseases) share similar patterns of invisibility, inequity in access to care and social isolation.

The findings revealed that most of the participants consistently emphasised that they either had to ‘fight’ to access the necessary healthcare services or were lucky to get them, particularly for psychological support and speech‐language therapy. Furthermore, the interviewed parents struggled to have their voices heard and emphasised the importance of viewing families not just as recipients of care, but as key partners and experts in their own right. This highlights a random equity or inequity of care as a central role in their experiences, as noted across multiple interviews, suggesting a shared perspective among participants.

### 4.1 Invisibility: An Inadequate Healthcare System and Policy

Our results show that parents of children with 22q11DS struggle to find experts in the field, particularly those who offer targeted psychological support. These findings align with the existing literature, as many families struggle to find healthcare professionals with a sufficient understanding of the syndrome's diverse, multisystem presentation [16]. In one study, almost all participating parents had previously encountered an HCP who was unfamiliar with 22q11DS, leading to a loss of trust in the professional's ability to offer appropriate and personalised care for their child [17]. A recent scoping review on the experiences of affected individuals emphasised the limited availability of specialised centres of excellence that regroup different experts for managing 22q11DS and highlighted the importance of multidisciplinary, integrated care [18, 19, 20].

The interviewees also expressed concerns about the higher medical costs associated with obtaining the needed support. Social insurance coverage or other types of public funding, where available, were not always enough to cover their children's needs (i.e. for psychotherapy or speech‐language therapy), and private funding was often too expensive. This corresponds to previous research [21] that identifies the financial burden as a major caregiving challenge for parents [22]. Financial inaccessibility highlights a gap in healthcare policies that fails to channel proper resources [10, 23]. More importantly, participants pointed out that resources did not always address the special needs of children and adolescents with 22q11DS. Funds attributed to disability care are always considered to be lacking [24]; In the literature on 22q11DS, however, it is the adolescents and young adults' population that's more subject to scarce resources (e.g., in the sexual health and transitional care systems [25]).

It is however important to keep in mind that healthcare coverage in the US is primarily based on a mix of private insurance and public programmes. In contrast, most European countries and Canada provide universal healthcare coverage through publicly funded systems, resulting in more equitable access to care. However, we did not aim to compare systems.

Additionally, most parents discussed the complex administrative procedures and the lengthy waiting lists to access health services. Both aspects have been mentioned in another study on 22q11DS, further highlighting the inadequate medical resourcing and the complex referral processes [12].

### 4.2 Inequity in Access to Care: Limited Physical Access and Access to Information

According to the parents, physical access to multidisciplinary or specialised clinics on 22q11Ds was limited and difficult to manage. This appears to be a common problem for families across the globe; in Brazil, many caregivers of individuals with 22q11DS travelled considerable distances to access health services, with 52.3% reporting travel requirements to receive care [21]. Interestingly, some participants in our study were relieved when their children were able to access support, such as psychotherapy or speech‐language therapy, during school hours, on‐site at the school. This seemed to ease their stress as these caregivers often struggle with time poverty due for example to the numerous medical consultations and therapies they need to take their children to [10, 26]. This is in fact the first study to discuss access to care (therapy) in 22q11DS through schools. Although this system already exists, for example, in some European countries, however, it is important to approach the distinction between practicality and quality of services with caution, and further in‐depth research is needed to examine the real‐world situation. Moreover, in terms of accessibility, caregivers interviewed described access to information as insufficient. This is consistent with other work in which parents of children with 22q11DS reported feeling uninformed about their child's condition due to a lack of accessible information [13] and assessed the lack of information on 22q11DS as one of the highest caregiving challenges [10].

### 4.3 Social Isolation: Lack of Awareness and Caregiver Support

In this study, the majority of parents described having had many encounters with HCPs unfamiliar with their child's condition. They also expressed concern that providers sometimes use stigmatising language, such as referring to their child as having something ‘wrong’ or focusing heavily on the child's disability and limitations rather than offering hope or emphasising the support and resources available to help the child flourish according to their capabilities. Moreover, parents were asked to almost objectify their children, to see them as a ‘challenge’, to focus on all those medical aspects and ‘detach themselves from the beautiful child that they have’, to see them as being in need of ‘special’ care and ask for it. This seemed hurtful and dehumanising, causing distress. This is consistent with the literature describing a lack of awareness of HCPs [10] (e.g., regarding the name Di George [12]). Similarly, parents sensed a lack of empathy from HCPs who sometimes perceived 22q11DS too negatively [12], leaving parents with a sense of their children being stigmatised.

The interviewees mentioned the impactful role of expanding the support to the caregiver and even to the family of a child with 22q11DS for their personal mental health. Many researchers have tackled the impact of 22q11DS on caregivers [22, 27, 28, 29, 30], addressing their heightened need for proper psychological support and a follow‐up on their mental health [10, 22] and how a caregiver's psychological distress was associated with a worsening of their child's mental health [31]. For example, one pilot study confirmed the acceptability of an online mindful parenting programme tailored to caregivers of children with medical complexities, including 22q11DS [32]. Our results showed that some parents had access to psychological support, but, compared to other research, psychological caregiver support was often financed by the personal caregiver's resources [13].

### 4.4 Practical Recommendations

The participants' experiences highlight specific areas in need of improvement. Drawing on the study's findings, several practical recommendations can be proposed to enhance support services (see Figure 2). Increased investment in healthcare services for children with 22q11DS, with particular emphasis on addressing the needs of both the affected individuals and their parents is recommended. Parents should be recognised as key partners in the care process and guided on how to access information (i.e. through social services in the hospital). Furthermore, a re‐evaluation of the administrative procedure to lower the administrative burden and the criteria of eligibility for support should be made to ensure a fair and equitable process. It is thus important to find ways to achieve the latter even with scarce resources. One solution includes having Centres of Reference for Rare Diseases (present in some European countries) that have positive impact on diagnosis and access [33] instead of having one for each rare disease. This could be combined with virtual case discussions where experts from these centres provide diagnostic and therapeutic advice to treating physicians [34]. And, although digitalisation was mentioned only in terms of telemedicine, however, the use of digital systems improves the quality of care as it reduces the number of times people are required to tell their story (i.e. through one central digital medical record) or ‘fill out forms’, especially if there is consensus on the standardisation of questionnaires for 22q11DS [35, 36, 37]. A digital system can also increase access to information for the families [38].

**Figure 2:**
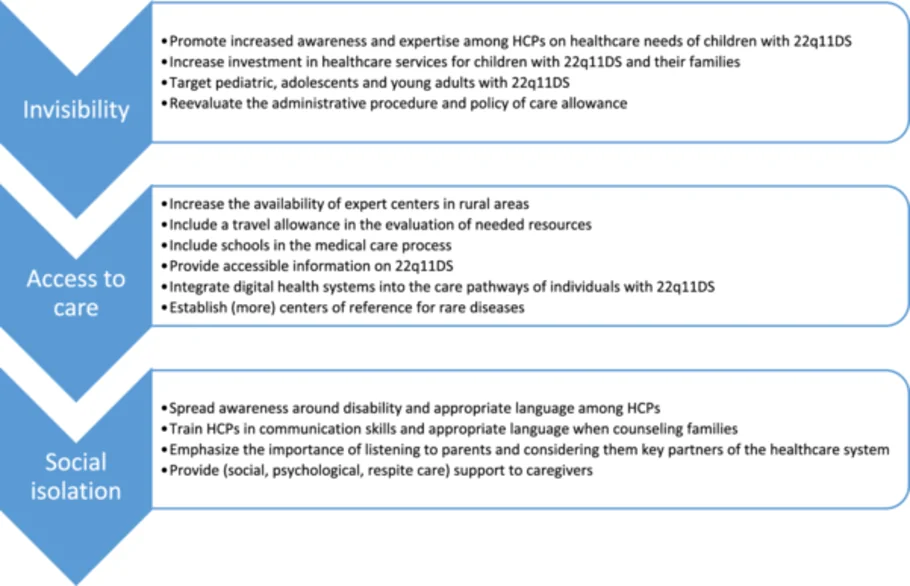
Practical recommendations for improving healthcare services for 22q11DS.

## 5 Limitations

The findings of this research are not generalisable to all families of children with 22q11DS, particularly because of differences in healthcare systems depending on the location. We also acknowledge a selection bias, as the parents who participated in the interview study are usually those with easier access to services and an interest in research. Additionally, most caregivers were mothers, and there is often a difference in perspective between mothers and fathers regarding healthcare, highlighting the difficulty of recruiting fathers in health research [39]. Finally, the study sample may reflect a degree of self‐selection as individuals with caregiving challenges might have been more inclined to take part, resulting in a heightened sensitivity to inequity and the gaps in the healthcare system. These limitations help identify areas where future research and support efforts could be most impactful.

## 6 Conclusion

Our study focused on the provision of healthcare services, using the AAAQ framework adapted to the context of 22q11DS. It revealed various challenges related to care for children with 22q11DS and their families, driven by limited access to specialised and empathic providers, lack of caregiver support and an underperforming healthcare system in terms of unequal resource allocation and complicated bureaucratic procedures. Harmful language and (ab)normality framing further undermine equitable care. Ensuring continuous healthcare support, including clear guidance on how and where to access information on healthcare services, is essential to improving the fairness and quality of services delivered. Healthcare providers should receive education extending beyond medical knowledge to include awareness of the importance of actively listening to parents, as well as addressing stigma and discrimination faced by individuals with disability in general, and individuals with 22q11DS particularly, and their families.

## Author Contributions

**Sophie Ayoub:** investigation, methodology, validation, visualisation, writing – review and editing, data curation, writing – original draft. **Sandra Meier:** conceptualisation, writing – review and editing, resources, project administration, funding acquisition. **Cheryl Cytrynbaum:** resources, writing – review and editing. **Ann Swillen:** resources, writing – review and editing. **Patricia Lingley‐Pottie:** writing – review and editing. **Andrea Shugar:** writing – review and editing. **Nicolas Graf:** writing – review and editing. **Holly Carbyn:** writing – review and editing. **Abiaz Hossain:** writing – review and editing. **Nathan Rowarth:** writing – review and editing. **Bernice S. Elger:** conceptualisation, funding acquisition, writing – review and editing, project administration, resources, supervision, validation, methodology. **Eva De Clercq:** methodology, conceptualisation, writing – original draft, writing – review and editing, supervision, validation, formal analysis.

## Ethics Statement

The study was approved by the Ethics Committee of the University of Basel for research with human subjects. The study was conducted in compliance with the protocol, the current version of the Declaration of Helsinki, the ICH‐GCP, and ISO EN 14155 (as far as applicable) as well as all national legal and regulatory requirements. All participants signed a written informed consent.

## Conflicts of Interest

The authors declare no conflicts of interest.

## Supporting information


Supporting File

## Data Availability

All data generated or analysed during this study are included in this published article and its supplementary information files.
