# Supplementary material for: ‘A Constant Battle for Services’: Parents' Experiences on Healthcare Services for Children With 22q11 Deletion Syndrome
Source: Health Expect. 2026 Oct 9;29(5):e70911. doi: 10.1111/hex.70911 (PMC13650500; doi:10.1111/hex.70911)
Supplement: Supplementary file 1 [file HEX-29-e70911-s001.docx]

**Interview Guide for parents**

| **Introduction** | Explanation regarding the project and why we find it important to address this topic. |
| --- | --- |
| **Narrative of the lived experience** | I am interested in your everyday life experience of being a parent of and caring for a child with 22q11.  While I have some questions to guide us along in the interview process, I am interested in hearing about anything else that you think would be useful to my understanding.  Then just one last reminder: there are no right or wrong answers; I am only interested in listening to what *you* have to say. |
| Interview guide: |  |
| **Ice-breaker**  **Pre-diagnosis**: On the time leading up to diagnosis  **Post-diagnosis**:  Care-giving experience  Support  Needs | **Before we begin, I would like to get to know your family a little better, so I know who you are talking about during the interview**.  **Can you tell me a bit about X (name of child with 22q11)?**  **Can you tell me a bit more about how you experienced the time leading up to your child’s diagnosis?**  **In caring for your child with 22q11 please describe to me:**   1) **Your everyday caregiving to the child** 2) **What you think and feel about caring for the child?** 3) **Please describe a recent experiences of child care that has made a deep impression upon you. Referring to this experience, what challenges or difficulties did you encounter in caregiving? How did you handle such challenges? Please illustrate your ideas with examples.**   **Can you walk me through the type of support you have received since your child has been diagnosed?**  **Are you satisfied with the support you have received since your child’s diagnosis?**  **Have the supports needs of your family and child changed over time (from childhood, to puberty)?**  **Can you describe me a bit in more detail the kind of needs you and your family** (including siblings) **have (had)?** |
| **Impact** | **How would you describe the impact of your child’s condition on your life immediately after the diagnosis was made?**  **How would you describe its impact on your life now?** |
| **Coping**  Experiences  Suggestions | **Can you tell me about what helps you coping?**  **Has your coping experience changed over time** (i.e. when your child was diagnosed compared to now or now your child is growing up)**? Can you explain a bit more in what way/or how?**  **Have you noticed any difference between your coping experience and that of your partner? If so, can you briefly talk about it?**  **What suggestions would you give to other parents to help them cope?** |
| **Present & Future** | **Now I would like to ask you to choose three adjectives or words that describe your current relationship with X (name of child). I know this may take a bit of time, so go ahead and think for a minute. Then I would like to ask you why you chose them**.  **Could you tell me about how you feel regarding the future?** **Your own future, that of your family and that of X (name child with 22q11)?** |
| **Closing questions**  **Intervention tool** | **How do you feel the community/society at large views your child’s condition/22q11?** (e.g. ignorance, stigma)  **We are developing/have developed an online coaching intervention to improve children’s wellbeing, enhance parental skills, and to reduce parental distress**. **What is your opinion regarding the availability of such a coaching program?**  **Is there anything that I have not asked about your experience and the support you have found or would like to find, that you would like to share?**  **How has talking with me today been like? Is there some way in which I could have made the interview easier/more comfortable for you?** |
